# Towards optimal design of anti-malarial pharmacokinetic studies

**DOI:** 10.1186/1475-2875-8-189

**Published:** 2009-08-06

**Authors:** Julie A Simpson, Kris M Jamsen, Ric N Price, Nicholas J White, Niklas Lindegardh, Joel Tarning, Stephen B Duffull

**Affiliations:** 1 Centre for Molecular, Environmental, Genetic and Analytic Epidemiology, School of Population Health, University of Melbourne, Melbourne, Victoria, Australia; 2 Mahidol-Oxford Tropical Medicine Research Unit, Faculty of Tropical Medicine, Mahidol University, Bangkok, Thailand; 3 International Health Program, Menzies School of Health Research and Charles Darwin University, Darwin, Northern Territory, Australia; 4 Centre for Clinical Vaccinology and Tropical Medicine, Churchill Hospital, Oxford, UK; 5 School of Pharmacy, University of Otago, Dunedin, New Zealand

## Abstract

**Background:**

Characterization of anti-malarial drug concentration profiles is necessary to optimize dosing, and thereby optimize cure rates and reduce both toxicity and the emergence of resistance. Population pharmacokinetic studies determine the drug concentration time profiles in the target patient populations, including children who have limited sampling options. Currently, population pharmacokinetic studies of anti-malarial drugs are designed based on logistical, financial and ethical constraints, and prior knowledge of the drug concentration time profile. Although these factors are important, the proposed design may be unable to determine the desired pharmacokinetic profile because there was no formal consideration of the complex statistical models used to analyse the drug concentration data.

**Methods:**

Optimal design methods incorporate prior knowledge of the pharmacokinetic profile of the drug, the statistical methods used to analyse data from population pharmacokinetic studies, and also the practical constraints of sampling the patient population. The methods determine the statistical efficiency of the design by evaluating the information of the candidate study design prior to the pharmacokinetic study being conducted.

**Results:**

In a hypothetical population pharmacokinetic study of intravenous artesunate, where the number of patients and blood samples to be assayed was constrained to be 50 and 200 respectively, an evaluation of varying elementary designs using optimal design methods found that the designs with more patients and less samples per patient improved the precision of the pharmacokinetic parameters and inter-patient variability, and the overall statistical efficiency by at least 50%.

**Conclusion:**

Optimal design methods ensure that the proposed study designs for population pharmacokinetic studies are robust and efficient. It is unethical to continue conducting population pharmacokinetic studies when the sampling schedule may be insufficient to estimate precisely the pharmacokinetic profile.

## Background

Despite significant progress in malaria control over the last few years, malaria-related morbidity and mortality are still considerable[1]. The contribution of inadequate dosage regimens (dose and frequency of administration) to anti-malarial treatment failure and the emergence of resistance has been underappreciated[2]. Most recommended dosage regimens are based on studies in non-pregnant adult patients, yet young children and pregnant women are at the greatest risk of treatment failure and often bear the brunt of the malaria burden[2]. Optimizing current treatment regimens could provide major health benefits and is a necessary prerequisite for malaria elimination. Effective treatment of malaria requires that the dose and frequency of administration of the anti-malarial drug provide drug concentrations over time (known as the 'concentration-time profile') sufficient to kill all of the parasites in the body. This profile is determined by the pharmacokinetic properties of the anti-malarial drug. In endemic areas, young children have the least immunity and the worst treatment responses. Anti-malarial pharmacokinetics often differ substantially between patients and, therefore, need to be quantified precisely for all key target populations, especially young children and pregnant women. A recent pharmacokinetic study for sulphadoxine-pyrimethamine (SP) found that children, receiving the same weight-adjusted SP dose as adults, had an area under the drug concentration-time profile, for both sulphadoxine and pyrimethamine, that was half that observed for adults [3]. The authors recommended that the current SP dose for children needs to be revised.

Over the last two decades developments in statistical methodology have allowed drug concentration-time profiles to be derived from patients providing only "sparse" samples at random times[4,5]. Data from all patients are modelled simultaneously using nonlinear mixed effects modelling (also known as 'population pharmacokinetic (PK) modelling'). This provides estimates of the mean population PK parameters (e.g. clearance), the inter-individual variability of the PK parameters, and the residual variability (which includes intra-individual variability, measurement errors and model misspecification). Additionally the introduction of liquid chromatography mass spectrometry has revolutionized drug analysis and is a highly sensitive assay with low limits of quantification while at the same time permitting low (i.e. 25–50 ml) sample volumes. The viability of field studies has been considerably improved by the introduction of sensitive assays using dried capillary whole blood spots taken on filter paper. Thus, since the PK profile can be determined from fewer blood samples per patient using nonlinear mixed effects modelling, and that the drug concentration can often be determined from blood taken by a simple finger prick, it has become easier to include pregnant women and young children in PK studies. PK studies, which determine the drug concentration-time profile in the target patient population, are known as 'population PK studies'. The importance of population PK studies in drug development and evaluation has been recognized by the US FDA[6].

Although there are now statistical methods to analyse sparse data from population PK studies, the design of these population PK studies is not straightforward. Many questions arise about how best to conduct these studies including: how many patients should be included in the study, what sampling scheme should be used, how many different sampling schemes are required, and how many patients need to be allocated to each of these sampling schemes. Answers to these questions require prior knowledge of the PK profile of the drug, the statistical methods used to analyse data from population PK studies, and also the practical constraints of sampling the patient population. Examples of such constraints include the timing of clinic visit, the maximum number of blood samples per patient that can be collected, logistical constraints of clinic workload, and whether a venous or capillary sample is being collected. Currently population PK studies are designed based on logistical, financial and ethical constraints, and the investigator's knowledge of the drug concentration-time profile. Although these factors are important, they may result in a study of 'insufficient design', which is unable to determine the desired PK parameters and, therefore, unable to answer the fundamental questions that the population PK model was intended to answer. In such a case the study is wasted. Thus, there is both a scientific and an ethical imperative to ensure PK studies are designed in such a way that they are most likely to provide useable results.

A prime example of the applicability, but also the difficulty of a population approach is seen with PK studies of the artemisinin derivatives. These compounds are rapidly absorbed and have very short elimination half-lives, typically less than one-hour [7], thus limiting options for sampling schedules. Additionally, there is large inter-patient and residual variability [8-13], reflecting in part issues with the previous drug assay methodologies [14]. Another complication is that these drugs decompose in the presence of iron(II) and, therefore, have to be analysed in plasma, and not in samples collected using the dried blood spot technique. This is because during the sample collection of dried blood spots, the blood cells rupture and the protein configuration is changed during the drying process. All of these factors may contribute to an 'insufficient design' for population PK studies of these drugs. Consequently, many of the PK analyses of the artemisinin derivatives have either been unable to fit satisfactory structural PK models to the data [9-11,13], fit only a one-compartment structural PK model [9,13], or required some of the PK parameters to be fixed [8,9,13]. In the study by Mithwani *et al *[9], the authors state that a number of assumptions were required to obtain a satisfactory fit of the model to the PK data due to the sparseness of the data. Considering that the key PK information required for the artemisinins is the maximum concentration, the area under the concentration-time profile, and the time above a certain concentration, then, it is unethical to continue conducting these PK studies when the sampling schedule will be insufficient to obtain accurately this information.

These various problems could be overcome by designing population PK studies using both information regarding the practical constraints of sampling and optimal design methods[15]. Optimal design methods provide designs for population PK studies that are robust and efficient by taking into account the concentration-time profile of the drug, the statistical methods required to analyse population PK data, practical constraints of sampling, and uncertainty in the PK parameter values and structural PK models. They can also help the researcher understand the benefits and limitations of particular sampling schedules.

This paper will provide an introduction to optimal design methods, including the prior information required for optimal design methods, followed by a theoretical application of the method for designing a population PK study of intravenous artesunate.

## Optimal design methods

Optimal design methods (also known as data-independent methods) for designing population PK studies have only been available in the last decade [15-17]. The methods have been used for designing studies of the population PK studies of itraconazole and ibuprofen in cystic fibrosis patients [18,19], enoxaparin in patients with acute coronary syndrome and deep vein thrombosis [20], mizolastine in children with allergies [21], and fluconazole in people with HIV infection[22].

The methods require prior specification of:

• structural PK model (e.g. one-compartment PK model with first-order absorption and elimination),

• PK parameter values (e.g. the values of the absorption rate constant (k_a_), clearance (CL), apparent volume of distribution (Vd)),

• the distribution of inter-individual variability for each of the PK parameters and the values of the variance of each of these distributions,

• the residual variability model and the values of the variance(s),

• study design space determined from sampling constraints (examples of sampling constraints: maximum number of patients that can be recruited in the study; maximum number of samples allowed per patient; minimum time allowed between consecutive samples for an individual; constrained times for the collection of the blood samples, say 9 am to 5 pm; cost limitations).

**Figure 1:**
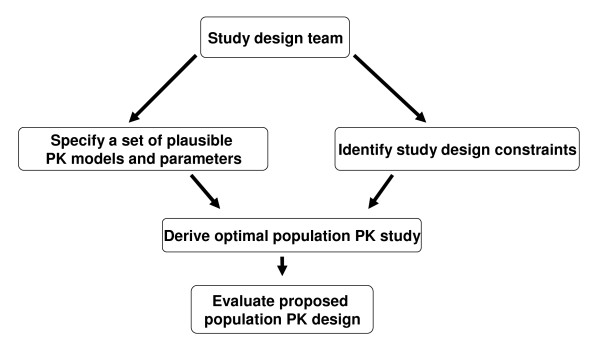
**Flowchart for designing population pharmacokinetic studies using optimal design methods**.

Figure 1 provides a flow-chart showing the components involved in designing a population PK study using optimal design methods. The study design team should have clinical, pharmacological and statistical expertise.

It is important to note that optimal design methods, when applied to PK studies, are dependent on the prior choice of a particular set of parameters and model. The influence of this dependence can be diluted by considering and accounting for uncertainty in both the parameter values as well as the plausible models. For example, Roos *et al *[22] used optimal design methods to determine a PK design for fluconazole in patients with HIV infection, using prior information from a phase I study of healthy volunteers. Since the prior information was from healthy volunteers who may differ in their PK of fluconazole, five different competing structural PK models were considered when designing the study for the patients with HIV infection. This ensured that the design proposed for HIV patients was robust to uncertainty in the structural PK model.

In addition to consideration of uncertainty in the prior information on the models, it is also important to consider that the optimum design may not necessarily be achievable in the clinic. Hence, it is important to consider practical constraints such that an optimum design is achieved conditioned on logistical components. An example using constraints on optimal design methods is given in [21]. In this work, a restrictive sampling schedule was developed for a population PK study of mizolastine in children. The study design constraints specified were a maximum of four and six blood samples drawn from a catheter in children aged 2 to 6 years and 6 to 12 years respectively, and only one sample from children aged 6 to 12 years without a catheter. For children with a catheter, blood sampling was restricted to the first six hours so they would not have to remain for a long time in the hospital. A population PK study, based on the derived optimal design, was then conducted in children and the corresponding data were successfully analysed using nonlinear mixed effects modelling.

Optimal design methods have not been used for designing population PK studies of anti-malarial treatments.

## Derivation of optimal design

To derive the optimum design, several candidate designs within the study constraints are considered, where the amount of "information" (i.e. the precision of the parameters to be estimated) in each design is estimated. Thus the design that contains the most "information" (i.e. the design with the most precision) is considered optimal. This is done mathematically by computing the Fisher information matrix based on parameters of the structural PK model, the inter-patient variability, and the residual variability. A brief explanation of the Fisher information matrix is given in Additional file 1. The design that maximizes the determinant of the Fisher information matrix (the determinant can be thought of as a measure of the volume of a matrix) also minimizes the determinant of the estimation variance-covariance matrix. In other words, optimizing a design based on the determinant of the Fisher information matrix will provide a design that has the highest precision (smallest standard errors) of the parameters to be estimated. The optimum design is called the D-optimal design. An optimization algorithm [23] searches for the D-optimal design within the study design constraints.

There are currently five open source software packages that derive optimal designs for population PK studies using the above D-optimal criteria, PFIM[24], PopDes[25], PopED[26], and POPT and its complement WinPOPT[27]. All of the packages evaluate sampling designs using the same mathematical derivation of the Fisher information matrix described above but vary in terms of the extensibility of the package [28]. For this paper, further discussion will be limited to the properties and features of the software package POPT (WinPOPT is the graphical user interface of POPT). POPT allows the user to specify various study constraints (maximum number of patients, maximum number of samples per patient, sampling windows, minimum time between consecutive blood samples). For prior specification of the structural PK model, the user can select one of the available PK models or specify their own model using any parameterization. Additionally, more than one structural PK model can be selected (e.g. one- and two-compartment models). For example, the structural PK model fitted to mefloquine concentrations in population PK studies has been both one-compartmental [29,30] and two-compartmental [31]. Since it is not known which model may best describe the data arising from the new study it is reasonable to consider all previously published models as potential candidates for the structural PK model. POPT then automatically combines a measure of the "informativeness" of the design for each PK model into a single criterion. This criterion can then be optimized using one of the global search algorithms available within POPT to provide a design that is jointly optimal for estimation of parameters from all candidate PK models.

Besides searching for the D-optimal design, POPT can evaluate the performance of any particular design. This informs researchers of the expected precision of the PK parameters and any limitations of their proposed population PK design.

## Application of the optimal design method to design a population pharmacokinetic study of intravenous artesunate

The following hypothetical example is a population PK study to determine the PK of dihydroartemisinin (the principal biologically active metabolite) following a single intravenous infusion (given as a rapid push) of 120 mg of artesunate (circa 2.4 mg/kg). It is assumed that artesunate is rapidly and completely converted to dihydroartemisinin. The study population are adults requiring intravenous treatment for malaria.

**Figure 2:**
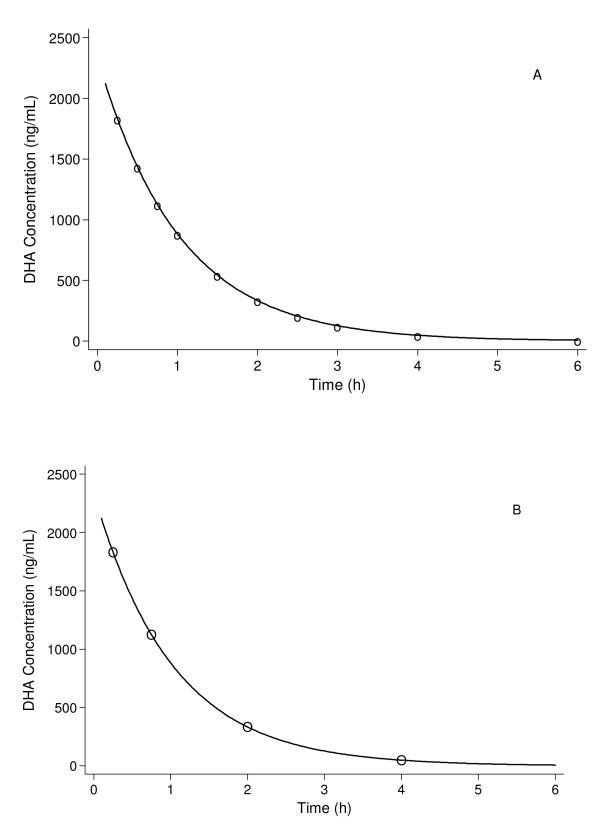
**Pharmacokinetic profile of dihydroartemisinin following a single intravenous dose of 120 mg artesunate (Vd = 38 L, CL = 37 L.hr^-1^)**. Superimposed on the pharmacokinetic profile is the symbol "o" (Panel A) to represent the sampling times for the rich design in Table 1 (20 patients, 10 samples per patient) and the symbol "O" (Panel B) to represent the sampling times for the sparse design in Table 1 (50 patients, 4 samples per patient).

Prior information regarding the structural PK model and PK parameters is required. For this example a one-compartment model with first-order elimination was selected to describe the PK profile of dihyroartemisinin:



The mean values chosen for *Vd *and *CL *were 38 L and 37 L.hr^-1^[32], and the PK profile simulated for these mean values is presented in Figure 2. These values were selected based on a preliminary review of literature. When designing an actual population PK study, the parameter values should be selected after an extensive literature review, which may provide a number of competing PK models and parameter sets. This review needs to account for sampling schemes and assay limitations, and adjust accordingly, as many of the differences between PK studies of anti-malarial drugs can be ascribed to differences in the assays or sampling schedule. This ensures that the final population PK design will have been optimized to provide good estimates of the parameters for all of the candidate PK models.

Inter-patient variability was assumed to be log-normally distributed with the inter-patient standard deviation equal to 0.5 for both *Vd *and *CL*, and covariance of *Vd *and *CL *equal to zero. Residual variability was assumed to be additive with a standard deviation of 200 ng.mL^-1^.

Sampling constraints were based on preliminary findings of a survey of 16 experts, who currently conduct anti-malarial PK studies in Asia and Africa. The constraints identified for venous sampling of an adult were: minimum time of 15 minutes between consecutive blood samples of a patient; and no more than 10 samples in the first 8 hours post drug administration.

The total number of patients to be recruited for our hypothetical population PK study is constrained to be 50 and the total number of blood samples that could be transported to a laboratory and assayed was fixed at 200.

Table 1 presents an evaluation of different sampling designs for a total of 200 blood samples. The four elementary designs presented illustrate how alterations in the number of patients and number of samples per patient affect the precision of the parameter estimates and efficiency of the design. All of the designs provide estimates of the parameters within the expected precision limit (expressed as 100 × standard error/estimate) of 15% for the PK parameters, less than 40% for the inter-patient variability, and less than 10% for the residual variability. The designs with more patients and less samples per patient improve the precision of the PK parameters and inter-patient variability, and improve the overall efficiency by at least 50%. The mixed design consisting of 10 patients with 10 samples per patient and 25 patients with 4 samples per patient, had slightly less expected precision for the PK parameters and inter-patient variability compared with the sparse design (50 patients with 4 samples per patient) but had marginally greater expected precision for the residual variability.

**Table 1:** Evaluation of elementary designs^a ^for population pharmacokinetic studies of adults with moderately severe malaria receiving a single dose of 120 mg intravenous artesunate

Number of patients	Number of samples per patient	Sampling times	Expected precision^b ^of PK parameter estimates (initial values^c^: *Vd *= 38 L, *CL *= 37 L.hr^-1^)	Expected precision^b ^of inter-patient variability estimates (initial values: ω_Vd _= 0.5, ω_CL _= 0.5)	Expected precision^b ^of residual variability estimate (initial value: σ_ε _= 200 ng.mL^-1^)	Efficiency^d ^(%)
20	10	{0.25, 0.5, 0.75, 1, 1.5, 2, 2.5, 3, 4, 6}	11.5, 11.4	33.5, 33.1	5.6	100 (criterion = 13.7)

40	5	{0.25, 0.75, 1, 2, 4}	8.2, 8.2	24.2, 24.3	6.5	157

50	4	{0.25, 0.75, 2, 4}	7.4, 7.6	21.8, 22.9	7.1	177

10	10	{0.25, 0.5, 0.75, 1, 1.5, 2, 2.5, 3, 4, 6}	8.8, 8.9	25.8, 26.6	6.2	143
25	4	{0.25, 0.75, 2, 4}				

^a ^– total number of blood samples constrained to be 200;

^b ^– expected precision expressed as 100 × standard error/estimate;

^c ^– Batty KT et al [30]; ω – sd of inter-patient variability (lognormal distribution);

^d ^– Relative comparison of criterion of sampling design to criterion of rich design of 20 patients (expressed as a percentage). The criterion is the determinant of the population Fisher information matrix raised to the power of one over the number of parameters to be estimated (for this example there are 5 parameters: *Vd*, *CL*, ω_Vd_, ω_CL_, σ_ε_)

These findings demonstrate the advantage of population PK studies compared with traditional PK studies of a small group of patients. The elementary designs presented have fixed sampling times for each of the patients. For a study in the field, which requires flexibility with blood sampling (for logistic reasons), POPT can generate optimal sampling windows around the fixed time points. Based on the fixed sampling times of 0.25, 0.75, 2 and 4 hrs for 50 patients presented in Table 1, the four sampling windows derived by POPT were 0.25–0.4, 0.6–1, 1.1–2.6, and 3–6 hrs. An evaluation of the sampling times at the boundaries of the sampling windows, observed at least 95% statistically efficiency of the design with the fixed time points.

The above elementary designs were evaluated and compared based on the expected precision (calculated using the Fisher information matrix) of the parameters, inter-patient and residual variability. Once a final design has been selected the design should also be evaluated by a population PK analysis of simulated data[22]. At least 100 datasets of virtual patients should be simulated that include uncertainty in the prior values of the PK parameters and execution error in the conduct of the trial.

## Conclusion

Population PK studies determine the drug concentration-time profile in the target patient population and the patient factors that cause changes in the drug concentration-time profile. These studies might be easier than traditional detailed PK studies to conduct, but they are complex to design and analyse. The design of a population PK study is very different to a sample size calculation for the number of participants required for a randomized controlled trial which can be performed using a hand calculator. Optimal design is necessary to ensure we design robust and efficient population PK studies that are able to estimate precise PK parameters. This is the necessary prerequisite for optimizing dose regimens. It is especially important for population PK studies of anti-malarials in pregnant women and young children in whom blood sampling cannot be extensive. Optimal design is a collaborative process involving clinicians, pharmacologists and statisticians. This research team plans to provide optimal designs for population PK studies of the artemisinin derivatives and their partner drugs. Prior specification of the structural PK model and parameters will be determined from an extensive literature review. Study design constraints for all malaria patients will be identified from the survey of clinicians and field workers actively involved in anti-malarial pharmacokinetic studies.

## Competing interests

The authors declare that they have no competing interests.

## Authors' contributions

JAS, SBD, RNP and NJW conceived the project. JAS wrote the first draft of the manuscript. KMJ, RNP, NJW, NL, JT and SBD revised the manuscript critically for important intellectual content. All authors read and approved the final manuscript.

## Supplementary Material

Additional file 1 Explanation of the Fisher information matrix. Click here for file
