# Supplementary material for: Towards optimal design of anti-malarial pharmacokinetic studies
Source: Malar J. 2009 Aug 6;8:189. doi: 10.1186/1475-2875-8-189 (PMC2732628; doi:10.1186/1475-2875-8-189)
Supplement: Additional file 1 — Explanation of the Fisher information matrix. [file 1475-2875-8-189-S1.pdf]

Additional file 1

Fisher information for an individual

To explain the Fisher information matrix, this example will start with the simple case of fitting a one compartment PK model to data for a single individual who received an intravenous infusion (given as a rapid push) of 120 mg artesunate. It is assumed that artesunate was rapidly and completely converted to the active metabolite, dihydroartemisinin (DHA). For simplicity, a bolus one-compartment PK model for DHA is assumed, since the infusion phase of artesunate and the conversion to DHA is very rapid. The model is given by

$$C_j = \frac{D}{Vd} \exp\left(-\frac{CL}{Vd} t_j\right) + \varepsilon_j, \tag{1}$$

where

- C_j represents the DHA concentration at time j,
- CL and Vd are the PK parameters clearance and volume of distribution, respectively,
- D is the artesunate dose of 120 mg multiplied by 284/384 to allow for the different molecular masses of artesunate and DHA, and
- ε_j represents the residual error, that is the difference between the predicted and observed DHA concentrations (C_j), and is assumed to have zero mean and an unknown variance σ_ε^2.

The Fisher information for data of a single individual is represented by the matrix

$$\boldsymbol{\mathcal{I}}(CL, Vd) \approx \boldsymbol{J}\boldsymbol{V}^{-1}\boldsymbol{J}^T, \tag{2}$$

where

- $\boldsymbol{J}$ is a p (no. of PK parameters) $\times$ n (no. of sampling times) matrix of first partial derivatives of the model with respect to the PK parameters at each sampling time,
- $\boldsymbol{V}$ is the $n \times n$ residual variance matrix which only has diagonal elements equal to σ_j^2, thus
- $\boldsymbol{\mathcal{I}}(CL, Vd)$ is a $p \times p$ matrix where the diagonal elements of its inverse represent the variance of the parameters, CL and Vd, according to Cramér-Rao inequality (for model (1) above, $\boldsymbol{\mathcal{I}}(CL, Vd)$ is a 2×2 matrix).

To illustrate, this example will evaluate a design of 4 sampling times at 0.25, 0.5, 1 and 2 hours post dosing to determine the PK parameter CL for an individual. It is assumed that Vd is known and fixed at 38 L, and σ_ε equals 200 ng.mL^{-1} (0.2 mg.L^{-1}) and is constant across all time points. So here $\boldsymbol{J}$ is a 1×4 matrix (one PK parameter, four sampling times):

$$\boldsymbol{J} = \left(\frac{\partial C_1}{\partial CL} \; \frac{\partial C_2}{\partial CL} \; \frac{\partial C_3}{\partial CL} \; \frac{\partial C_4}{\partial CL} \right),$$

where

$$\frac{\partial C_j}{\partial CL} = -\frac{t_j D}{Vd^2} \exp\left(-\frac{CL}{Vd} t_j\right) \quad \text{for } j = 1, \ldots, 4.$$

Also, $\boldsymbol{V}^{-1}$ is just $\boldsymbol{I}_4 \times (1/\sigma_\varepsilon^2)$, where $\boldsymbol{I}_4$ is the 4×4 identity matrix. Thus based on the above values and four sampling times the Fisher information is

$$\boldsymbol{\mathcal{I}}(CL) \approx \boldsymbol{J}\boldsymbol{V}^{-1}\boldsymbol{J}^T = \frac{1}{0.2^2} \sum_{j=1}^{4} \left(-\frac{t_j 120(\frac{284}{384})}{38^2} \exp\left(-\frac{CL}{38} t_j\right)\right)^2,$$

which yields a single value (since CL is the only PK parameter). In words, the above Fisher information is the sum of the information at the four sampling times: 0.25, 0.5, 1 and 2 hours post dosing, weighted by the residual variance (0.2^2). Based on a prior value for CL of 37 L.hr^{-1},

$$\boldsymbol{\mathcal{I}}(CL) \approx 0.0337.$$

Thus the expected variance of CL equals 29.67 (1/0.0337), giving a standard error of 5.45 L.hr^{-1}. This can be expressed as a coefficient of variation of 14.73%.

Fisher information for a population PK study

For a population PK study there are DHA concentration data from many individuals, and a statistical model is fitted to the data of all individuals simultaneously using nonlinear mixed effects modelling. The model for the single individual (1) is now extended to include an additional level of variability for each parameter, known as the inter-individual variability:

$$\begin{aligned} C_{ij} &= \frac{D}{Vd_i} \exp\left(-\frac{CL_i}{Vd_i} t_{ij}\right) + \varepsilon_{ij} \qquad (3) \\ CL_i &= CL \exp(\eta_{CL,i}) \\ Vd_i &= Vd \exp(\eta_{Vd,i}). \end{aligned}$$

So now,

- C_{ij} represents the jth DHA concentration for the ith individual,
- CL and Vd are the population mean clearance and volume of distribution, respectively,
- CL_i and Vd_i represent the i_{th} individual PK parameters and are assumed to be lognormally distributed across individuals,
- the η_i's represent the differences between the ith individual PK parameters and the population mean PK parameters, and are assumed to have zero mean and variance-covariance matrix $\boldsymbol{\Omega}$ (with variances ω_{CL}^2 and ω_{Vd}^2 on the diagonal), which represents the inter-individual variability for the parameters, and
- ε_{ij} represents the residual error, that is the difference between the predicted and observed DHA concentrations (C_{ij}), and is assumed to have zero mean and an unknown variance σ_ε^2.

The Fisher information matrix for individual i with a set of sampling times (denoted ξ_i) is derived from the log likelihood function of model (3), where the model is approximated using a first-order Taylor expansion around the expectation of the inter-individual variability [14]. The resulting Fisher information matrix is a block diagonal matrix, where the first block contains the information for the population means (CL and Vd), and the second contains the information for the inter-individual and residual variability ($\omega^2_{CL}, \omega^2_{Vd}$, and σ^2_ε). In fact, the Fisher information matrix given in the last section is just a special case of this - there was only one element of the first block (CL). Please refer to [14] for the formal derivation of the Fisher information matrix for nonlinear mixed-effects models.

The *population* Fisher information matrix is then just the sum of the Fisher information matrices derived for each individual. Here it is assumed that all individuals (N) are sampled at the same time points, hence all individual Fisher information matrices are exactly the same (implying $\xi_1 = \xi_2 = \cdots = \xi_N$). Let $\boldsymbol{\Psi}$ be the vector of all estimable parameters in model (3), i.e. $\boldsymbol{\Psi} = (CL, Vd, \omega^2_{CL}, \omega^2_{Vd}, \sigma^2_\varepsilon)$ and $\boldsymbol{\Xi}$ be the set of all individual sampling schedules $(\xi_1, \xi_2, \cdots, \xi_N)$. The population Fisher information matrix can then be expressed as

$$\boldsymbol{\mathcal{I}}(\boldsymbol{\Psi}, \boldsymbol{\Xi}) = \sum_{i=1}^{N} \boldsymbol{\mathcal{I}}(\boldsymbol{\Psi}, \xi_i) = N \cdot \boldsymbol{\mathcal{I}}(\boldsymbol{\Psi}, \xi_i). \tag{4}$$

To illustrate, the example is expanded to evaluate a design of the same sampling times (0.25, 0.5, 1 and 2 hours) but for 30 individuals instead of a single individual. As above, it is assumed that Vd is known and fixed at 38 L, and σ_ε equals 200 ng.mL^{-1} (0.2 mg.L^{-1}) and is constant across all time points. Since this study now has 30 individuals, it is also assumed that the inter-individual standard deviation for Vd is known and fixed at 0.5.

The evaluation was performed in `POPT` using the prior value for CL of 37 L.hr^{-1}, and a prior value for the inter-individual standard deviation for CL (ω_{CL}) of 0.5. The expected standard error for CL was 3.56, leading to a coefficient of variation of 9.61%. Thus the increased number of individuals has increased the precision for CL. Additionally, `POPT` gives the expected standard error for ω^2_{CL}, which was 0.07, leading to a coefficient of variation of 28.70%.
